# Programmed Obesity?: Study Links Intrauterine Exposures to Higher BMI in Toddlers

**Published:** 2009-01

**Authors:** M. Nathaniel Mead

To date, there have been relatively few epidemiologic studies investigating the association between intrauterine exposure to chemicals and body mass index (BMI, which characterizes weight in relation to height). Now a prospective birth cohort study in Flanders, Belgium, reveals an association between prenatal exposure to environmental pollutants and elevated BMI during the first three years of life **[*EHP* 117:122–126; Verhulst et al.]**. The study also found associations between exposures and birth weight and length.

Since the late 1990s, developmental biologists have amassed laboratory data indicating that exposure to endocrine disruptors such as polychlorinated biphenyls (PCBs), dioxins, and bisphenol A during critical phases of fetal development may increase the risk of obesity later in life. In addition, prenatal exposure to cigarette smoke has been linked with the subsequent development of obesity. These exposures likely alter mechanisms involved in weight homeostasis.

Using a longitudinal study design, the researchers obtained a random sample of 138 mother–infant pairs by drawing from 26 maternity wards in Flanders between September 2002 and February 2004. These wards were identified within geographic areas with varying environment and pollution characteristics (rural, urban, and industrial). The researchers collected information on the parents that included health status, smoking behavior, age, family composition, socioeconomic status, height, and weight. Children’s height and weight were also analyzed, and umbilical cord blood was collected to enable measurement of levels of hexachlorobenzene, dioxin-like compounds, PCBs, and the pesticide metabolite DDE. Children were then observed over a 3-year period.

The main findings were twofold. First, higher PCB congener levels were associated with higher BMI standard deviation scores (SDS) in the children between 1 and 3 years of age. Both maternal smoking and higher PCB levels were positively associated with birth weight SDS. In addition, they showed a statistically significant combined effect. Second, higher DDE levels were associated with a slight increase in BMI SDS in 3-year-old children of nonsmoking mothers, but this effect was further increased in children of smoking mothers. Thus, simultaneous intrauterine exposure to endocrine disruptors might compound the weight-enhancing effects of maternal smoking during pregnancy.

The researchers acknowledge a number of study limitations, including the fact that maternal weight gain during pregnancy—an important risk factor for obesity in children—was not recorded in the study. Additionally, the children were only followed for 3 years. Although it remains to be seen whether BMI trends for this particular study population will persist later in life, a higher BMI during toddlerhood is known to be associated with an increased risk of obesity in adulthood.

This is the first epidemiologic study to demonstrate the impact of prenatal pollution exposure on BMI during the preschool years, and more large-scale prospective studies are certainly warranted. One research challenge will be to determine which factors mediate the effects of prenatal exposure to endocrine disruptors in terms of subsequent weight dysregulation in the offspring.

## Figures and Tables

**Figure:**
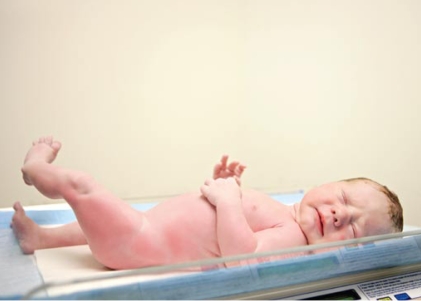
Certain exposures in utero can affect birth weight, birth length, and later, body mass index.

